# Caregiver-Centered Communication: Engaging Family Caregivers

**DOI:** 10.1093/geroni/igaa057.1890

**Published:** 2020-12-16

**Authors:** George Demiris, Karen Hirschman

## Abstract

Older adults are often relying on a family member or other informal caregiver (friend or other) to jointly navigate the health care system and cope with the ramifications of serious illness; thus, the patient-caregiver dyad becomes the unit of care. The caregiving role becomes crucial in cases where patients are facing a condition that limits their cognitive and functional abilities and caregivers are called to act as proxy decision makers for significant treatment and symptom management decisions. Caregivers often report that they feel isolated and overwhelmed, and in some cases experience significant barriers in communicating with health care providers. It is important that clinicians communicate in a way that acknowledges and addresses caregivers’ preferences, needs and perspectives. Caregiver centered communication can facilitate a more effective adaptation throughout the illness course with better adherence to recommended treatment plans and greater satisfaction with care for both patients and families, as well as a more comprehensive response to their psychosocial needs. While health care organizations often aim to increase caregiver engagement and involvement in care processes, there is a lack of tools or strategies not only to more actively engage caregivers but also to assess how ongoing approaches perform in terms of facilitating meaningful and inclusive communication. This symposium will review existing tools and a new instrument to measure caregiver centered communication, challenges and opportunities in measuring the quality of communication with caregivers and highlight empirical data of communication quality in various health care settings including home care and hospice.

